# Prediction of outcome following paraquat poisoning by arterial lactate concentration-time data

**DOI:** 10.3892/etm.2014.1773

**Published:** 2014-06-11

**Authors:** LIANG SUN, GUO-QIANG LI, PENG-BO YAN, YANG LIU, GUO-FENG LI, LU-QING WEI

**Affiliations:** Department of Respiratory and Critical Care Medicine, Pingjin Hospital, Logistics University of the Chinese People’s Armed Police Forces, Tianjin 300162, P.R. China

**Keywords:** arterial lactate, paraquat, poisoning, prognosis

## Abstract

The present study retrospectively analyzed 170 patients diagnosed with paraquat (PQ) poisoning with the aim of clarifying whether the arterial lactate-time (arterial lactate concentration × time between ingestion and arterial lactate measurement) was a good predictor of mortality in patients with acute PQ poisoning. The results indicated that there was a positive correlation between the arterial lactate-time and PQ concentration-time (ρ=0.485). In addition, the arterial lactate-time data exhibited a similar discriminative power to the plasma PQ concentration-time data (z=0.712; P=0.864). For the receiver operating characteristic curve analysis, the lactate-time data had an area of 0.782 with a cut-off value of 11.95 mmol/l.h (sensitivity, 64.52%; specificity, 84.42%). To calculate the predicted probability of survival for any specified time and initial arterial lactate concentration, the following formula was derived based on the logistic regression coefficients: Logit(p) = 3.066 − 0.139 × (time lag following PQ ingestion) − 0.177 × (initial arterial lactate concentration); where the probability of survivors = 1/1 + e^−logit(p)^. Therefore, the arterial lactate-time data exhibited a good predictive power for evaluating the prognosis of patients with acute PQ poisoning.

## Introduction

Paraquat (PQ) is a widely-used herbicide that has been shown to cause severe and often fatal pulmonary fibrosis in humans and laboratory animals in developing countries, such as China and Sri Lanka. Following the ingestion of large amounts of concentrated formulation, the rapid development of multi-organ failure and cardiogenic shock is almost universally fatal (1,2). Even with the ingestion of smaller amounts, PQ is actively taken up into pulmonary epithelial cells where redox cycling and free radical generation triggers a fibrotic process that may lead to mortality. Management of PQ poisoning has remained predominantly supportive and the results for the treatment of PQ poisoning, including immunosuppressive therapy (3,4), prolonged extracorporeal elimination (5) and lung transplantation, have been disappointing (6,7).

A reliable predictor of prognosis may guide treatment and future clinical research on antidotes and other therapies (8). The measurement of plasma PQ concentration has been considered as a marker of severity and prognosis (1). Proudfoot *et al* produced a nomogram in 1979 that indicated a correlation between the outcome to the plasma PQ concentration on admission with the time from ingestion to blood collection. Patients with PQ levels lower than a line connecting concentrations of 2.0, 0.6, 0.3, 0.16 and 0.1 mg/ml at 4, 6, 10, 16 and 24 h, respectively, have been shown to survive (9). Scherrmann *et al* identified that 30 survivors following PQ poisoning had plasma PQ levels of >C mg/ml, where C = 1/[0.471 × time (h) since ingestion × 1.302] (10). The studies by Hart *et al* (11) and Gil *et al* (12) demonstrated a very strong correlation between measured concentrations and survival rates. In addition, the correlation between the urine PQ concentration and the time following ingestion has also been used to evaluate the prognosis of patients with PQ intoxication (13). However, a major constraint of these methods is the inability of numerous hospitals in the developing world, where the majority of patients present with this condition, to perform the required assay. An additional important predictor of mortality is the quantity of PQ consumed. However, estimates on the amount ingested are often unobtainable or unreliable in a number of intoxicated patients.

Recently, the serum lactate level has been used as a prognostic marker in patients with acute PQ poisoning, and higher arterial lactate levels have been shown to be associated with a higher risk of mortality through multiple logistic regression [odds ratio (OR), 7.02; 95% confidence interval (CI), 2.06–23.91; P=0.002] (14). In an additional study, receiver operating characteristic (ROC) curve analysis revealed that the initial arterial lactate concentration had an area of 0.749 (95% CI, 0.714–0.856) and a cut-off level of 2.5 mmol/l for the prediction of prognosis in patients with acute PQ poisoning (15).

However, initial arterial lactate concentrations may vary, even in patients that have ingested the same amount of PQ, since blood samples are collected at different times. Therefore, it has been hypothesized that the initial arterial lactate concentration alone is not sufficiently predictive to infer effectiveness. However, initial arterial lactate concentration-time data may have a better predictive power compared with initial arterial lactate concentration alone. Thus, the aim of the present study was to assess initial arterial lactate concentration-time data as a prognostic marker of mortality and morbidity in patients with PQ intoxication.

## Materials and methods

### Patients and setting

The study protocol was reviewed and approved by the Clinical Trial Committee of Pingjin Hospital (Tiajin, China). Informed consent was provided by the patients or by their next of kin prior to therapy.

The study included 170 patients with PQ poisoning. The time lag following PQ ingestion and the arterial lactate and plasma PQ concentrations were measured at the same time following admission. However, not all the patients had their urine PQ concentration measured quantitatively, as a dithionite method was applied, which provided a qualitative determination. Demographic variables, including age and gender, were recorded in all the patients, and in particular, whether mortality occurred during the stay at hospital. All the patients who survived to the time of discharge were visited after six months to determine if there had been any delayed mortalities.

This retrospective observational study occurred in an 18-bed poisoning Emergency Treatment Center of the University affiliated Pingjin Hospital between June 2008 and June 2012. Blood samples were collected on admission and the arterial lactate levels were measured with a blood gas analyzer (GEM premier 3000; Instrumentation Laboratory, Bedford, IL, USA) immediately following admission. Quantitative analysis of the plasma samples from all the PQ-poisoned patients were conducted in the hospital laboratory using a gas chromatography method (16). Patients who met any of the following criteria were excluded from the study: Non-oral ingestion poisoning, admission to a different hospital or PQ exposure of >24 h previous to presentation.

### Treatment

To prevent the absorption of PQ by the gastrointestinal tract, gastric lavage was performed via a nasogastric tube using 1 g/kg activated charcoal in 500 ml saline (0.9%) once every 4 h. In addition, SMECTA (Beaufour Ipsen Pharmacy Co., Ltd., Tianjin, China) and magnesium sulfate powder (Tianjin Huairen Pharmacy Co., Ltd., Tianjin, China) were placed into 20% mannitol (Shuanghe Pharmaceutical Co., Ltd., Tianjin, China), which was administered rectally. All patients received activated charcoal hemoperfusion therapy (Braun Diapact CRRT machine; B Braun Medical, Ltd., Hesse, Germany), followed by 12 h continuous veno-venous hemofiltration (CVVH) therapy with a 4 h interval following hemoperfusion. During the 4 h interval and following CVVH, the patients received high-dose therapy comprising an intravenous infusion of 15 mg/kg/day cyclophosphamide (Guangdong Qingping Pharmacy Co., Ltd., Guangzhou, China) in 250 ml glucose saline (5%) for 1 h for two days and 1 g/day methylprednisolone sodium succinate injection (Pfizer, Inc., New York, NY, USA) iin 250 ml glucose saline (5%) for 2 h for three days. Starting on day 4, patients also received intravenous injections of 5 mg dexamethasone (Jilin Extrawell Changbaishan Pharmaceutical Co., Ltd., Jilin, China) every 6 h. In addition, vitamin E capsules (Xinyi Pharmaceutical Co., Ltd., Shanghai, China), metoprolol (AstraZeneca, London, UK) and vitamin E injections (Zhongjing Biotechnology Co. Ltd., Harbin, China) were administered.

### Statistical analysis

SPSS statistical software package 20.0 (IBM, Armonk, NY, USA), MedCalc 12.4 (MedCalc Software, Ostend, Belgium) and GraphPad Prism v 4.0 (GraphPad Software, Inc., La Jolla, CA, USA) were used to perform statistical analysis. Data are presented as the mean ± standard deviation or as the median with the range. Statistically significant differences between the two groups were analyzed using the independent two-sample t-test or the Mann-Whitney U-test. Arterial lactate concentration, arterial lactate-time and PQ concentration-time data were compared using ROC curve analysis to analyze the statistical significance of the differences between the areas under the ROC curve, according to the method by DeLong *et al* (17). The cut-off values were determined by analyzing the Youden’s index and the maximized area under the ROC curve.

In addition, multiple logistic regression analysis was performed with log transformed data to derive a formula that had the best predicted survival rates of the study population. Multiple logistic regression analysis of the initial parameters focused on mortality using a backward elimination method.

## Results

### Baseline characteristics

A total of 170 patients were enrolled in the study and the baseline characteristics of the patients are described in Table I. Of the 170 subjects, there were 97 females and 73 males, and 93 survivors and 77 patients that succumbed to acute PQ intoxication. In all the patients, the average time interval between PQ ingestion and the first sample collection was 6.46 h (range, 2.5–19 h). However, the median times for the survivors and non-survivors were 5.00 (range, 4.00–8.00 h) and 6.00 h (range, 4.00–8.75 h), respectively. Non-survivors exhibited a higher average arterial lactate concentration of 5.00 mmol/l (range, 2.00–10.00 mmol/l) and a plasma PQ concentration of 10.0 mg/l (range, 6.00–15.00 mg/l) when compared with the survivors that had arterial lactate and PQ concentrations of 2.00 mmol/l (range, 1.00–2.50 mmol/l) and 5.00 mg/l (3.00–7.00 mg/l), respectively.

### ROC curve analysis

With regard to the ROC curve analysis (Table II), the arterial lactate concentration had an area of 0.774 and the cut-off value was 4.2 mmol/l (sensitivity, 82.80%; specificity, 63.64%; Youden’s index, 0.464). The arterial lactate-time data had an area of 0.782 with a cut-off value of 11.95 mmol/l h (sensitivity, 64.52%; specificity, 84.42%; Youden’s index, 0.490). Positive correlations were observed between initial arterial lactate and plasma PQ concentrations (ρ=0.414), as well as between arterial lactate-time and PQ concentration-time (ρ=0.485; Table III). Results from the multiple logistic regression analysis of the initial parameters on mortality using a backward elimination method are described in Table IV. The results indicated that increased lactate concentrations (OR, 0.838; 95% CI, 0.755–0.930; P<0.001) were associated with a significantly higher risk of mortality when the time lag following PQ ingestion and PQ concentration for the two patients was equal.

**Figure 1:**
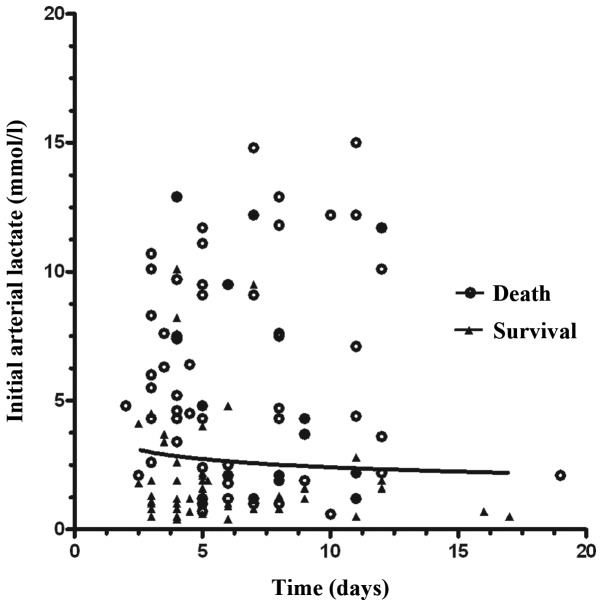
Correlation between the initial arterial lactate concentration and the time of ingestion for 170 patients with acute PQ poisoning. PQ, paraquat.

A logarithmic plot of the initial arterial lactate concentration against the time since ingestion is show in Fig. 1. To calculate the predicted probability of survival for any specified time and initial arterial lactate concentration, the following formula was derived based on the logistic regression coefficients: Logit(p) = 3.066 − 0.139 × (time lag after PQ ingestion) − 0.177 × (initial arterial lactate concentration); where the probability of survival = 1/1 + e^−logit(p)^.

**Figure 2:**
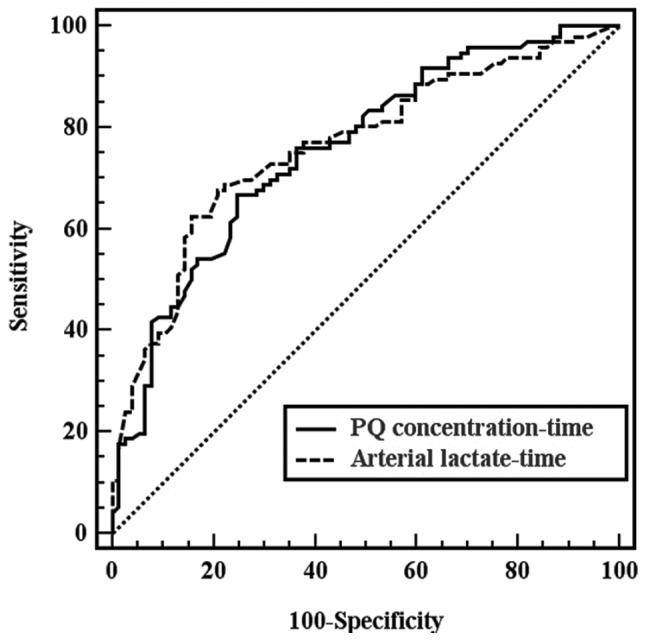
ROC curves for arterial lactate concentration-time and PQ concentration-time. Pairwise comparison of the ROC curves revealed that there was no significant difference between the areas under the curve for arterial lactate concentration-time and PQ concentration-time (z=0.712; P=0.864). ROC, receiver operating characteristic; PQ, paraquat.

Pairwise comparison of the ROC curves in Fig. 2 demonstrated that there was no statistically significant difference between the areas for arterial lactate-time and PQ concentration-time (z=0.712; P=0.864).

## Discussion

Increased blood lactate levels have been associated with significant morbidity and mortality since their first description in 1843 by Scherer (18). A number of studies have emphasized the prognostic importance of measuring a single lactate level during treatment (19). Despite this strong and already long-lasting predictive power of lactate levels, little evidence exists on the prognosis of acute PQ poisoned patients. Recently, a number of studies found that the arterial lactate concentration had predictive power for the prognosis of acute PQ poisoned patients (14,15). The results of the present study revealed that the arterial lactate concentration was not only higher in non-survivors (average, 5.00 mmol/l; range, 2.00–10.00 mmol/l) compared with survivors (average, 2.00; range, 1.00–2.50 mmol/l; P<0.001), but also exhibited a positive correlation with the PQ concentration (ρ=0.414). These observations may validate the hypothesis that the arterial lactate level possesses a good predictive power in evaluating the prognosis of patients with acute PQ poisoning.

However, initial arterial lactate concentrations may vary, even in patients that have ingested the same amount of PQ, since blood samples are collected at different times. The time following PQ ingestion should be considered as a marker of severity and prognosis. As shown in Table IV, logistic regression analysis, using time and arterial lactate concentration as variables, produced very significant independent effects for each variable as a predictor of survival. Therefore, we hypothesized that the initial arterial lactate concentration-time data may have a better predictive power compared with the initial arterial lactate concentration alone.

Plasma PQ concentration-time data have been used as a practical tool to predict the prognosis of acute PQ poisoned patients. The present study demonstrated that there was a positive correlation between the arterial lactate-time and PQ concentration-time (ρ=0.485). The arterial lactate-time data exhibited a similar discriminative power to the plasma PQ concentration-time data (z=0.712; P=0.864), thus, arterial lactate-time data may have a discriminative power as a practical tool in predicting the prognosis of acute PQ poisoned patients. To calculate the predicted probability of survival for any specified time and initial arterial lactate concentration, the following formula was derived based on the logistic regression coefficients: Logit(p) = 3.066 − 0.139 × (time lag after PQ ingestion) − 0.177 × (initial arterial lactate concentration); where the probability of survivors = 1/1 + e^−logit(p)^. The use of the logistic regression equation allows the prediction of the probability of survival for any specified time and initial arterial lactate concentration following the ingestion of PQ for ≤19 h.

The present study reports a novel correlation between the initial arterial lactate concentration and time, which may aid the prediction of patient survival following the ingestion of PQ for ≤19 h (Fig. 1). The logistic regression equation together with the survival curve (Fig. 1) may produce highly significant independent effects for each variable to predict the probability of survival. However, the new survival curve requires prospective validation to determine the sensitivity and specificity for predicting the outcome in patients with acute PQ poisoning.

There were several limitations in the present study. Firstly, this was a retrospective study; thus, a limited amount of quality data were collected. Secondly, since the times of mortality for the non-survivors were not collected, survival analysis of the arterial lactate concentration and arterial lactate-time was unable to be performed. Finally, the results may have been more informative if the urine PQ concentrations had been measured.

In conclusion, the arterial lactate-time data had a better predictive power compared with the arterial lactate concetration alone for evaluating the prognosis of patients with acute PQ poisoning. Therefore, measuring the initial arterial lactate concentration and the time of poisoning may be a simple and practical tool for assessing the severity of PQ poisoning.

## Figures and Tables

**Table I:** Demographic and laboratory observations of the survivors and non-survivors among the 170 patients with acute PQ poisoning.

Parameter	Survivors (n=93)	Non-survivors (n=77)	P-value
Age (years)	30.00 (23.00, 45.5)	29.00 (24.00, 46.50)	0.518
Time lag after PQ ingestion (h)	5.00 (4.00, 8.00)	6.00 (4.00, 8.75)	0.117
Arterial lactate (mmol/l)	2.00 (1.00, 2.50)	5.00 (2.00, 10.00)	<0.001
Arterial lactate-time (mmol/l.h)	10.00 (5.00, 17.50)	26.10 (15.00, 53.55)	<0.001
PQ concentration (mg/l)	5.00 (3.00, 7.00)	10.00 (6.00, 15.00)	<0.001
PQ concentration-time (mg/l.h)	26.50 (13.50, 47.50)	59.00 (35.50, 104.50)	<0.001

Results are expressed as median (range) values. PQ, paraquat.

**Table II:** Prediction of the mortality rate in acute PQ poisoning.

Parameter	Cut-off point	Sensitivity (%)	Specificity (%)	AUC (95% CI)	Youden’s index
Arterial lactate (mmol/l)	4.20	82.80 (73.57, 89.83)	63.64 (51.89, 74.30)	0.774 (0.703, 0.834)	0.464
Arterial lactate-time (mmol/l.h)	11.95	64.52 (53.91, 74.17)	84.42 (74.36, 91.68)	0.782 (0.712, 0.841)	0.490
PQ concentration (mg/l)	9.35	86.02 (77.28, 92.34)	59.74 (47.94, 70.77)	0.765 (0.694, 0.826)	0.462
PQ concentration-time (mg/l.h)	22.70	44.01 (32.79, 53.69)	92.21 (83.81, 97.09)	0.768 (0.697, 0.829)	0.362
Time lag after PQ ingestion (h)	6.50	66.67 (56.31, 75.96)	48.05 (36.52, 59.74)	0.568 (0.490, 0.644)	0.145

Results are expressed as median (range) values. PQ, paraquat; AUC, area under the curve; CI, confidence interval.

**Table III:** Correlation analysis between arterial lactate and PQ concentrations, measured at the same time after acute PQ poisoning.

Parameter	Statistical parameter	PQ concentration	PQ concentration-time
Arterial lactate (n=170)	Correlation coefficient (ρ)	0.414	
	P-value	<0.001	
Arterial lactate-time (n=170)	Correlation coefficient (ρ)		0.485
	P-value		<0.001

PQ, paraquat.

**Table IV:** Multiple logistic regression analysis of the initial parameters on mortality following PQ ingestion.

Parameter	OR	95% CI	P-value
Time lag after PQ ingestion	0.869	0.774, 0.978	0.002
Arterial lactate	0.838	0.755, 0.930	<0.001
PQ concentration	0.856	0.794, 0.923	<0.001

PQ, paraquat; CI, confidence interval; OR, odds ratio.
